# Pathological Observations

**Published:** 1831-04-01

**Authors:** 


### PATHOLOGICAL OBSERVATIONS. By *John Houston*, M.R.I.A.

[Dublin Hospital Reports, Vol. V.]

THESE pathological memoranda are short and miscellaneous, such as may be transcribed from the note-book of a practitioner—and not the less valuable on that account! If we had more of these plain records of facts, and less of the recondite speculations which are often based on no facts at all—or on false facts, medical science would be a gainer by the change! But it is needless to hope for any such millennium in our art.

### I. MALFORMATION.

A female was delivered of a male child, which exhibited no outward imperfection. On every attempt to suck, there supervened fits of suffocating cough, and the milk was all disgorged. An attempt was made to pass a bougie down the œsophagus, but failed. The infant died the next day. On dissection, the pharynx was found to terminate in a cul-de-sac, someway down the neck, without having any communication with the œsophagus. The larynx was perfect. "The trachea was also complete on the anterior and lateral parts; but the posterior wall was perforated by a wide opening, from which the œsophagus took origin, and which appeared so smooth and oblique as to render the passage from the larynx into that tube, as direct and easy as that along the trachea into the lungs."

It is hardly necessary to observe that, as soon as the milk accumulated in this cul-de-sac so as to rise on a level with the rima glottidis, and enter that chink, the symptoms of suffocation ensued. A drawing of the parts is given by the author.

### II. ATTEMPT AT SUICIDE.

In March, 1828, a servant attempted to destroy himself by cutting his throat with a razor. Mr. H. saw him ten minutes afterwards, and found him almost lifeless. Circulation and sense were suspended. The wound was frightfully deep, and extended almost from ear to ear. The razor had entered between the os hyoides and thyroid cartilage, disuniting them so completely as to allow the *former* to ascend with the tongue into the mouth. The pharynx was laid wide open, and the epiglottis severed from its attachment to the tongue and os hyoides, and thus left hanging by its pedicle to the back of the Pomum Adami. The carotids had escaped untouched, and the hemorrhage was inconsiderable. It appeared difficult at the moment to account for the sudden extinction of life. The symptoms were evidently those of suffocation, but the cause was not, at first, understood.

"I passed my finger into the wound, and found, to my surprise, that the epiglottis, loosened from its upper and lateral attachments, had fallen back over the rima glottidis, and completely intercepted the passage of air to the lungs. I raised the obstructing body and drew it forwards: the chest soon after began to heave, respiration returned, the heart and pulse again beat, and consciousness and sensibility were re-established.

It required some effort of my fingers to hold up the epiglottis, as the air at every inspiration tended to force it back again to its unnatural and dangerous position." 316.

In this dilemma the top of the epiglottis was brought over the edge of the thyroid cartilage and there secured by a ligature. The man, in a short time, sat up, but was unable to articulate. He was taken to the MEATH HOSPITAL, but never recovered from the delirium under which he laboured at the time of the suicide. He died a week after the attempt.

### III. EFFECTS OF THE ROPE IN HANGING.

In two individuals brought into the College of Surgeons for dissection, the appearances were so similar that our author deems them deserving of record.

"Both the individuals were strong plethoric men, executed for murder of a tradesman of their own craft under the combination system. Their death was evidently caused by strangulation. The cervical vertebræ were unbroken, and the spinal marrow and brain presented no marks of injury. In both the sterno-mastoid on the right side (the opposite to that on which the knot of the rope was applied) were ecchymosed, contused, and broken; those of the left side were only slightly bruised. The os-hyoides and thyroid cartilage were completely severed from each other. The omo-hyoid, sterno-hyoid, and thyro-hyoid muscles were so bruised and lacerated, that only some stretched shreds of them remained to hold the parts together. The thyro-hyoid membrane was also torn across, and the epiglottis, pulled from its root at the back of the thyroid cartilage, had passed up with the os-hyoides and tongue into the back part of the mouth; the skin alone remained unbroken, and interposed between the rope and the cavity of the pharynx. This was the only region of the neck which gave evidence of much injury; the great vessels and nerves all escaped unhurt.

In two other criminals, which more lately I have had an opportunity of examining, precisely the same effects had been produced by the rope on the soft parts of the neck, and without any injury to the brain or spinal marrow." 318.

We agree with Dr. Houston that the knowledge resulting from the *pathological* effects of hanging is not of much importance in medicine. The *moral* effects of this operation are of more consequence; but these are beyond our pale.

On this account also, we shall pass over the "case of a man who lost his life from having swallowed a large spoon." It is a thousand to one that any of our readers shall ever meet with such a case—and if they did, the effects of this suicidal performance would not be of the least use to them. The man died of peritonitis, and on dissection, the spoon was found lying across the spine, in the cavity of the duodenum, with one end of it in the pyloric orifice of the stomach. It had produced ulceration of the duodenum, with extravasation of its contents into the cavity of the abdomen.

### IV. INTERNAL STRANGULATION OF THE BOWELS AFTER PARTURITION.

This case is so short, and so incapable of abbreviation, that we must give it in the words of the author.

"A lady about 28 years of age, and the mother of several healthy children, to all of whom she had given birth without any untoward symptoms, was delivered of a healthy boy, her last child.

During her pregnancy she had experienced no unusual inconvenience, but immediately after the birth of the infant she complained of severe pain in the abdomen, which became violently aggravated on the placenta being discharged. No evacuations could be procured from the bowels; the belly became swollen and tense, and the pulse remarkably quick and small. She sunk rapidly under symptoms of the most acute peritonitis, and notwithstanding the most energetic treatment, expired in about thirty-four hours from the time of delivery.

*Dissection.* The uterus had contracted, and nearly retired into the pelvis. The entire peritoneal surface exhibited all the marks of intense inflammation, and the ileum, to the extent of about three feet, was completely sphacelated, black, and filled with blood. A band of lymph, two inches long, and of considerable thickness and solidity, was attached by one end to the right ovarium and fallopian tube, and by the other formed a tight noose around the mesentery of the mortified gut, which thoroughly strangulated and deprived it of circulation. The extravasation of blood, which had taken place into the cavity of the intestines, and among its tunics, marks the tightness of the ligature.

It would appear, from the absence of any disagreeable symptoms during pregnancy, and the rapidity and violence with which they followed the birth of the infant, aggravated still more after the discharge of the placenta, that the adhesion between the ovarium and mesentery had been contracted while the uterus was high in the abdomen, *and that the descent of that organ after parturition drew tight the band which strangulated the bowels.*" 322.

V. Two cases of malformation of the heart ensue, in which there was a communication between the black and red blood. In one of them the blue colour only came on when the little patient laboured under any pectoral affection. In the other, life was destroyed by repeated convulsions, at the age of 18 months. In both cases, there did not appear to be much inconvenience from the malformation, when there was no agitation of the system by disorder or strong exercise. We published, some years ago, a remarkable example of this kind, where the foramen ovale was quite open, but where there was no blue colour of the lips or skin except when the little patient was excited or irritated.

VI. ENCYSTED HYDROCEPHALUS. A healthy-looking infant was affected the day after its birth with slight convulsions, which increased, in frequency and severity, to the sixth week. They then ceased; but the child became more stupid, and the head began to enlarge. Audition appeared to be unimpaired, as also the sense of taste; but the eyes seemed insensible to light. When a year old, the head was enormously enlarged—the anterior fontanelle broad—and fluctuation was perceptible all through the cranium. At intervals the child voided large quantities of urine, followed by sensible diminution of size in the head, and alleviation of stupor. The bowels were always costive, and sleep was almost continued. It died at the age of 23 months, and the weight of the head was then equal to that of the whole body. We shall give the dissection in the words of the author.

*Dissection.* "The bones of the cranium were thin and flexible; the membranes of the brain healthy. The cerebral matter was soft, pale, and exsanguineous. The convolutions were nearly effaced, their surfaces broader than natural, and their interstices less deep. On the removal of the membranes the brain could with difficulty be supported entire, owing to the quantity of fluid in its interior. In the situation of the corpus callosum a transparent membrane existed, scarcely concealing the fluid which lay underneath. The lateral ventricles were vastly dilated, and thrown into one common cavity by the absorption of their natural partition, the septum lucidum, no vestige of which was discoverable; they were lined by a firm opaque membrane, smooth on the surface next the fluid, formed into folds, which divided the cavity into several lodges, and supplied with numerous blood-vessels larger and more turgid than those on the surface of the convolutions. The fluid which the ventricles contained was limpid, of a pale straw colour, and in great quantity. The cerebellum was reduced to one-half its usual bulk, its substance softened, and its convolutions scarcely traceable. Its place was occupied by the water of the fourth ventricle; and the arachnoid membrane, covering its posterior and lateral surfaces, was universally adherent to that lining the dura mater of the posterior fossa. The pons varolii was small, and soft in texture. The nerves, at their points of exit from the skull, were perfectly distinct, though their origins from the brain could with difficulty be traced.

So far the morbid anatomy of the case offers nothing very singular; the most remarkable circumstance connected with it remains yet to be noticed, viz., the existence of various sized cysts in different parts of the cerebral substance, unconnected with the ventricles, and which continued to hold fluid after the evacuation of water from these cavities. There were two such cysts, each of the size of an almond, in the site of the fornix and optic thalami. Two others were found in the anterior lobe of the right hemisphere, each capable of holding from one to two ounces of water, and perfectly distinct from each other and from the neighbouring ventricle. These cysts were distended with fluid and lined with membranes, bearing the same general characters as those which existed in the ventricles. There were two other small cysts near the base of the brain, analogous in their contents and in the qualities of their lining membrane to the proper ventricular cavity. The cerebral matter in the immediate neighbourhood of these cysts had undergone no material change, and differed not in quality from that in other more remote parts of the brain." 330.

---

